# Pulmonary tumor thrombotic microangiopathy induced by gastric carcinoma: Morphometric and immunohistochemical analysis of six autopsy cases

**DOI:** 10.1186/1746-1596-6-27

**Published:** 2011-03-30

**Authors:** Yoichiro Okubo, Megumi Wakayama, Kanako Kitahara, Tetsuo Nemoto, Tomoyuki Yokose, Fumihito Abe, Nobuyuki Hiruta, Daisuke Sasai, Minoru Shinozaki, Haruo Nakayama, Takao Ishiwatari, Kazutoshi Shibuya

**Affiliations:** 1 Department of Surgical Pathology, Toho University School of Medicine, 6-11-1, Omori-Nishi, Ota-Ku, Tokyo, 143-8541, Japan; 2 Department of Pathology, Kanagawa Cancer Center, 1-1-2, Nakao, Asahi-Ku, Yokohama-city, Kanagawa, 245-0815, Japan; 3 Department of Dermatology, Toho University Sakura Medical Center, School of Medicine, 564-1, shimoshizu, Sakura-city, Chiba, Japan; 4 Department of Neurosurgery, Toho University Ohashi Medical Center, 2-17-6, Ohashi, Meguro, Tokyo, 153-8515, Japan

## Abstract

**Background:**

Pulmonary tumor thrombotic microangiopathy (PTTM) has been known as a rare and serious cancer-related pulmonary complication. However, the pathogenesis and pathophysiology of this debilitating condition still remains obscure and no effective management was recommended. The present study aims to elucidate the pathophysiology of PTTM.

**Methods:**

Autopsy records were searched to extract cases of pulmonary tumor embolism induced by metastasis of gastric carcinoma in the Toho University Omori Medical Center from 2000 to 2006. And then, tissue sections of extracted cases were prepared for not only light microscopic observation but morphometric analysis with the use of selected PTTM cases.

**Results:**

Six autopsies involved PTTM and clinicopathological data of them were summarized. There was a significant negative association between pulmonary arterial diameter and stenosis rate in four cases. Although all cases showed an increase of stenosis rate to some degree, the degree of stenosis rate varied from case to case. Significant differences were found for average stenosis rate between the under 100 micrometer group or the 100 to 300 micrometer group and the 300 micrometer group in four cases. However, no significant differences were found for average stenosis rate between the under 100 micrometer group and the 100 to 300 micrometer group in all cases. Meanwhile, all cases showed positive reactivity for tissue factor (TF), five showed positive reactivity for vascular endothelial growth factor (VEGF), and three showed positive reactivity for osteopontin (OPN).

**Conclusions:**

In the present study, we revealed that the degree of luminal narrowing of the pulmonary arteries varied from case to case, and our results suggested that pulmonary hypertension in PTTM occurs in selected cases which have a widespread pulmonary lesion with severe luminal narrowing in the smaller arteries. Furthermore, our immunohistochemical examination indicated that gastric carcinoma indicating PTTM shows a higher TF-positive rate than typical gastric carcinoma. However, it remains still obscuring whether gastric carcinoma indicating PTTM shows a higher VEGF or OPN-positive rate as determined by immunohistochemistry.

## Background

Pulmonary tumor thrombotic microangiopathy (PTTM) is known as a rare and severe devastating cancer-related pulmonary complication that was first reported by Von Herbay et al. in 1990 [1]. Previous studies aimed to determine how this unique complication developed with metastasis of carcinoma cells. Some candidates produced by carcinoma cells were especially investigated as a key factor of the disease, such as tissue factor (TF) [2-7], vascular endothelial growth factor (VEGF) [2-7], and osteopontin (OPN) [7], and their contribution to the development of PTTM was investigated. Clinically, patients with PTTM often present with subacute respiratory failure with pulmonary hypertension, right-side heart failure, and sudden death [2-6]. PTTM is histopathologically characterized by tumor embolism, multiple microthrombi, and intimal myofibroblast proliferation in the pulmonary arteries and arterioles [1,2]. However, the pathophysiology of this debilitating condition still remains obscure. The present study aims to elucidate the pathophysiology of PTTM using morphometric and immunohistochemical examinations of six autopsy cases induced by metastasis of gastric carcinoma.

## Methods

### Patients and clinicopathological data selection

Autopsy records were searched to extract cases of pulmonary tumor embolism induced by metastasis of gastric carcinoma in the Toho University Omori Medical Center from 2000 to 2006. And then, paraffin-embedded pulmonary tissue sections of these cases with pulmonary tumor embolism induced by metastasis of gastric carcinoma were prepared, and stained with hematoxylin and eosin (HE) double stain and Elastica van Gieson (EVG) stain to determine whether they satisfied the diagnostic criteria of PTTM, histopathologically. For an accurate histopathological diagnosis, tissues were examined by more than three pathologists. In addition, we extracted and sampled data from the medical records associated with each case, such as age, sex, maximum diameter of the tumor, macroscopic type, histopathological type, lymph-vascular involvement, organs with metastasis, and clinical symptoms.

### Morphometric analysis of pulmonary arteries

Initially, all arterial images of pulmonary EVG-stained sections were photographed using a digital camera. In the present study, pulmonary arterial diameter, area within the internal elastic lamina (IELA), and lumen area (LA) were measured with Image J 1.36b software (National Institutes of Health, Bethesda, MD) using these photographed images. To evaluate the degree of pulmonary arterial stenosis, we calculated the stenosis rate using following formula: [1-(LA/IELA)] × 100 = stenosis rate (%).

Furthermore, we focused on PTTM cases with pulmonary hypertension. Therefore, we divided pulmonary arteries into three groups according to the Heath and Edwards classification [8], such as artery under 100 μm, 100 to 300 μm, or over 300 μm in diameter (designated the 'under 100-μm' group, the '100-300-μm' group, and the 'over 300-μm' group, respectively).

To allow a more detailed discussion of PTTM, we performed the following three investigations in each case. First, to assess any potential relationship between pulmonary arterial diameter and stenosis rate, we made a scatter plot of pulmonary arterial diameter and stenosis rate, and Spearman's rank correlation coefficient was calculated. Second, to clarify the frequency of pulmonary arterial lesion, the optional stenosis rate in each arterial group was calculated. Finally, we calculated the average stenosis rate in each arterial group and statistical significance was tested using the one-way analysis of variance (ANOVA) and the Bonferroni post hoc test. Differences were considered significant at P < 0.05.

### Immunohistochemical examination

In the present study, the gastric and pulmonary tissue sections in each case were used for immunohistochemical examination. Polyclonal anti-VEGF-antibody (Human vascular endothelial growth factor (polyclonal antibody), dilution 1:50; Immuno-Biological Laboratories Co., Gunma, Japan), monoclonal anti-TF-antibody (Human tissue factor, 1:100; American Diagnostica Inc., Stamford, United States of America), and anti- OPN-antibody (OP3N, dilution 1:100; Leica Biosystems Newcastle Ltd., Newcastle, United Kingdom) were used.

## Results

### Incidence of PTTM in our institute

578 autopsies were retrieved from the autopsy records of Toho University Omori Medical Center for the period from 2000 to 2006. According to these records, it was deemed that 37 patients died of gastric carcinoma and a total of six cases involved pulmonary tumor embolism induced by metastasis of gastric carcinoma. According to the diagnostic criteria of PTTM as proposed by Herbay et al. [1], we regarded these six autopsies as PTTM.

### Summary of patient characteristics

Patient age ranged from 49 to 82 years (mean ± standard deviation (SD): 68.3 ± 11.3). The gender ratio was 4:2 (male to female). Maximum diameter of the tumor ranged from 20 to 70 mm (mean ± SD: 45.8 ± 25.4). Chemotherapy was given in one case (Case 5). Transthoracic Doppler echocardiography was used to confirm the presence or absence of pulmonary hypertension in two cases (Case 1 and 2, respectively). As a result, case 1 and 2 involved clinically proven or denied pulmonary hypertension, respectively. Unfortunately, no appropriate examinations were performed in the other patients (Case 3 to 6) and it is not known whether they suffered from pulmonary hypertension [Table 1].

**Table 1:** Summary of patient characteristics

Case	Age	Sex	Maximum diameter of tumor (mm)	Macroscopic subtype	Histopathological subtype	Lymph-vascular involvement	Metastasis	Pulmonary arterial pressure
1	76	Male	70	Borrmann 3	por	ly-3 v-2	Lung, diaphragm, pleura, peritoneum, liver, gallbladder, pancreas, adrenal gland, lymph nodes.	90 mmHg
2	82	Female	20	Borrmann 2	por	ly-3 v-1	Lung, liver, pancreas, lymph nodes	10 mmHg
3	67	Female	30	Superficial depressed type	por	ly-3 v-1	Lung, esophagus, thyroid, pericardium, ovary, lymph nodes	Unknown
4	67	Female	75	Borrmann 3	por	ly-3 v-1	Esophagus, duodenum, ileum, jejunum, colon, mesenterium, liver, gallbladder, pancreas, kidney, adrenal gland, aorta, lymph nodes	Unknown
5	49	Female	60	Borrmann 3	pap	ly-3 v-2	Esophagus, duodenum, liver, pancreas, kidney, adrenal gland, lymph nodes	Unknown
6	72	Male	20	Borrmann 2	por	ly-3 v-1	Pancreas, spleen, kidney, adrenal gland, colon, spinal cord, lymph nodes	Unknown

por: poorly differentiated adenocarcinoma, pap: papillary adenocarcinoma, ly/v-1: minimal lympho-vascular involvement, ly/v-2: moderate lympho-vascular involvement, ly/v-3: prominent lympho-vascular involvement

Patient age ranged from 49 to 82 years (mean ± standard deviation: 68.3 ± 11.3). The gender ratio was 4:2 (male to female). Maximum diameter of the tumor ranged from 20 to 70 mm (mean ± standard deviation: 45.8 ± 25.4). Main histology of gastric carcinoma involved poorly differentiated adenocarcinoma in five of the six cases (Case 1, 2, 3, 4, and 6), while the remaining case involved papillary adenocarcinoma (Case 5). Although all cases more or less showed lymph-vascular involvement, lymphatic involvement was prominent. Case 1 and 2 involved clinically proven and denied pulmonary hypertension, respectively.

### Histopathological status of surgically obtained gastric carcinoma

**Figure 1:**
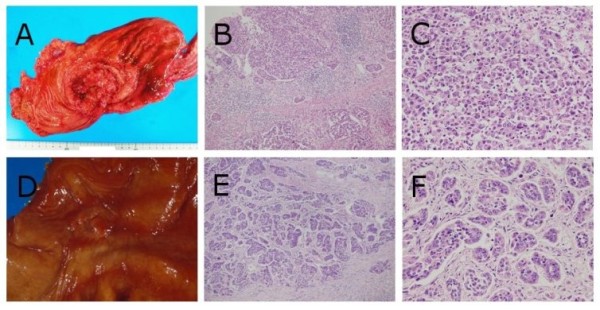
**Macroscopic and histopathological findings of gastric carcinomas in case 1 and 2**. Case 1 (A) The ulcerated tumor with relatively demarcated and raised margins (Borrmann's type 3 tumor) was located in the gastric body. (B and C) Poorly differentiated adenocarcinoma cells diffusely proliferated (hematoxylin-eosin (HE) double stain, × 100, × 400, respectively). Case 2 (D) The ulcerated tumor with demarcated and slightly raised margins (Borrmann's type 2 tumor) was located in the gastric body. (E and F) Poorly differentiated adenocarcinoma cells were arranged in small nests or trabecular pattern surrounded by fibrous stroma (HE double stain, × 100, × 400, respectively).

In the present study, the main histology of gastric carcinoma involved poorly differentiated adenocarcinoma (Case 1, 2, 3, 4, and 6), while the remaining case involved papillary adenocarcinoma (Case 5). Although all cases more or less showed lymph-vascular involvement, lymphatic involvement was prominent. Furthermore, the autopsies revealed that no patients had latent carcinomas [Figure 1 and Table 1].

### Morphometric analysis of altered pulmonary arteries

**Figure 2:**
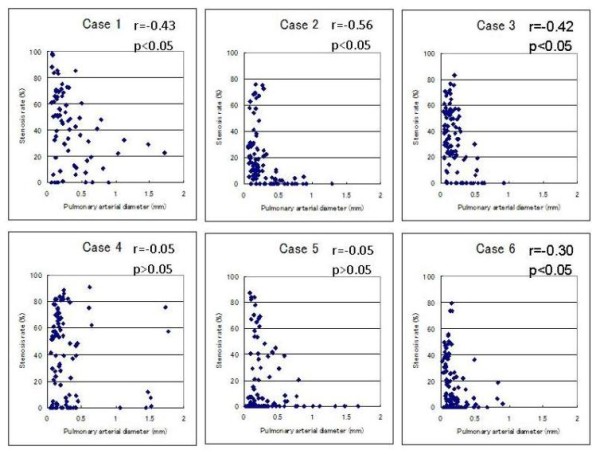
**Scatter plots of pulmonary arterial diameter and stenosis rate in pulmonary arteries**. There was a significant negative association between pulmonary arterial diameter and stenosis rate in four of the six cases (Case 1, 2, 3, and 6). In contrast, there was no significant association in the remaining two cases (Case 4 and 5).

**Figure 3:**
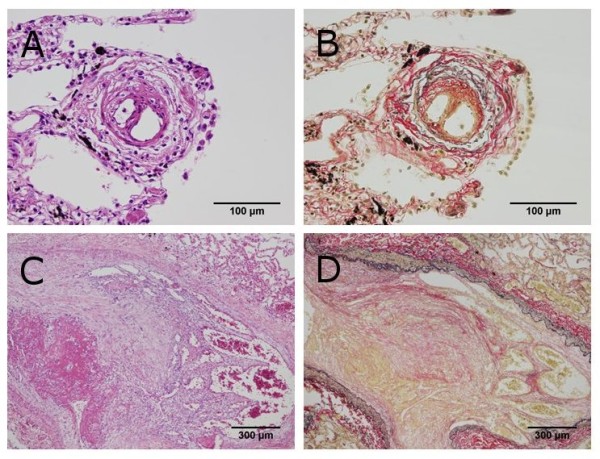
**Histopathological findings of pulmonary tumor thrombotic microangiopathy in case 1**. (A and B) Fibrocellular intimal proliferation occludes the lumen of the pulmonary artery under 100 μm in diameter (Hematoxylin-eosin (HE) double stain and elastica van Gieson (EVG) stain, × 400). (C and D) Fibrocellular intimal proliferation associated with carcinoma cells proliferation occludes the lumen of the pulmonary artery over 300 μm in diameter (HE double stain and EVG stain, × 40).

**Figure 4:**
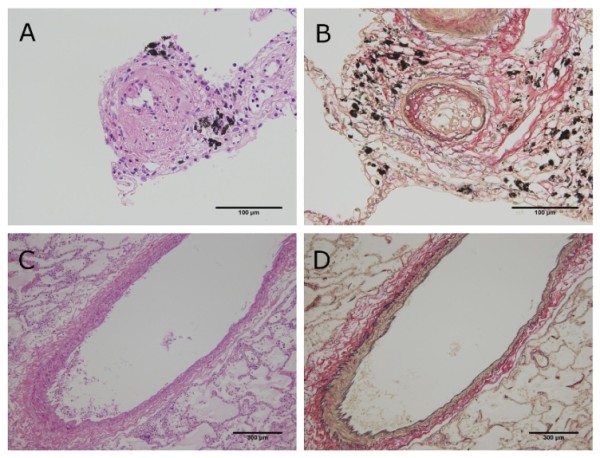
**Histopathological findings of pulmonary tumor thrombotic microangiopathy in case 2**. (A and B) Fibrocellular intimal proliferation occludes the lumen of the pulmonary artery under 100 μm in diameter (Hematoxylin-eosin (HE) double stain and elastica van Gieson (EVG) stain, × 400). (C and D) Neither prominent fibrocellular intimal proliferation nor carcinoma cells were present in the pulmonary artery over 300 μm in diameter (HE double stain and EVG stain, × 40).

**Figure 5:**
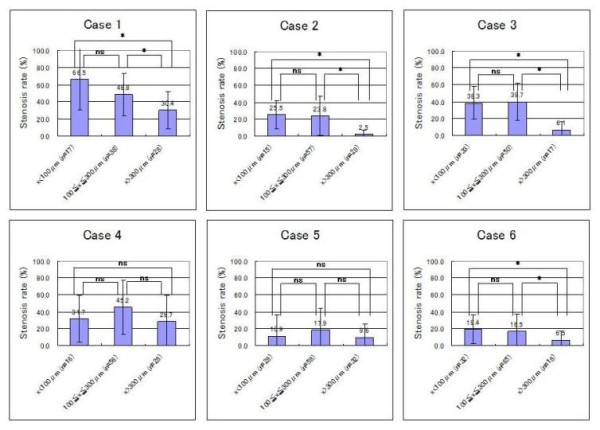
**Average stenosis rate in each arterial group for six patients with pulmonary tumor thrombotic microangiopathy**. No significant differences were found for average stenosis rate between the under 100-μm group and the 100 to 300-μm group in all cases. On the other hand, significant differences were found for average stenosis rate between the under 100-μm group or 100 to 300-μm group and the 300-μm group in four of the six cases (Case 1, 2, 3, and 6). Statistical significance was tested using the one-way analysis of variance (ANOVA) and the Bonferroni post hoc test. Values are expressed as mean ± standard deviation. Differences were considered significant at P < 0.05 (*: P < 0.05, ns: P > 0.05).

First, the scatter plot of pulmonary arterial diameter and stenosis rate and the result of the Spearman correlation coefficient are shown in Figure 2. According to the Spearman correlation coefficient, there was a significant negative association between pulmonary arterial diameter and stenosis rate in four of the six cases (Case 1, 2, 3, and 6). In contrast, there was no significant association in the remaining two cases (Case 4 and 5). Second, the relationship between optional stenosis rate and each arterial group is summarized in Table 2. Although all cases showed an increase of stenosis rate to some degree, the degree of change in stenosis rate varied from case to case [Figure 3 and 4]. Finally, no significant differences were found for average stenosis rate between the under 100-μm group and the 100 to 300-μm group in all cases. On the other hand, significant differences were found for average stenosis rate between the under 100-μm group or 100 to 300-μm group and the 300-μm group in four of the six cases (Case 1, 2, 3, and 6) [Figure 5 and Table 3].

**Table 2:** The relationship between optional stenosis rate and each arterial group

Case	Arterial group	Stenosis rate (%)			
		Over 50%	Over 40%	Over 20%	Over 10%
1	under 100-μm	76.5	76.5	76.5	76.5
	100 to 300-μm	60.5	68.4	81.6	86.8
	over 300-μm	10.7	35.7	64.3	82.1

2	under 100-μm	13.3	13.3	60.0	86.7
	100 to 300-μm	0.0	21. 1	38.6	64.9
	over 300-μm	0.0	0.0	3.4	6.9

3	under 100-μm	30.3	48.5	84.8	87.9
	100 to 300-μm	38.0	52.0	84.0	88.0
	over 300-μm	0.0	0.0	11.8	23.5

4	under 100-μm	43.8	50.0	62.5	62.5
	100 to 300-μm	57.1	57.1	69.6	73.2
	over 300-μm	25.0	35.7	46.4	50.0

5	under 100-μm	9.4	12.5	12.5	12.5
	100 to 300-μm	19.0	22.4	31.0	34.5
	over 300-μm	0.0	10.7	28.6	28.6

6	under 100-μm	0.0	12.5	50.0	59.4
	100 to 300-μm	9.2	16.9	29.2	38.5
	over 300-μm	0.0	0.0	11.1	22.2

Although all cases more or less showed an increase of the stenosis rate, the degree of change in stenosis rate varied from case to case. In particular, case 1 showed widespread severe stenosis in the pulmonary artery.

**Table 3:** The average stenosis rate in each arterial group (%)

Case	under 100 μm	100 to 300 μm	over 300 μm
1	66.5 ± 36.3	48.8 ± 24.9	30.4 ± 21.8
2	25.5 ± 16.7	23.8 ± 23.1	2.5 ± 4.8
3	38.3 ± 19.6	39.7 ± 22.0	6.1 ± 9.7
4	31.7 ± 27.4	45.1 ± 31.8	28.7 ± 30.9
5	10.9 ± 25.6	17.9 ± 26.9	9.6 ± 15.5
6	19.4 ± 16.8	16.4 ± 21.0	6.5 ± 10.1

The pulmonary arteries were divided into three groups according to the Heath-Edwards classification, such as artery under 100 μm, 100 to 300 μm, or over 300 μm in diameter (designated the 'under 100-μm' group, the '100 to 300-μm' group, and the 'over 300-μm' group, respectively). This table surmised the average stenosis rate in each arterial group. Values are expressed as mean ± standard deviation.

### Immunohistochemical examination

**Figure 6:**
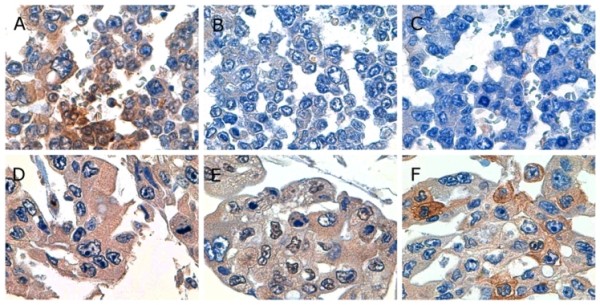
**Photomicrographs showing representative immunohistochemistry**. (A, B, and C) Gastric carcinoma cells in case 1 showed positive reactivity for tissue factor and negative reactivity for vascular endothelial growth factor and osteopontin (× 1,000). (D, E, and F) Gastric carcinoma cells in case 6 showed positive reactivity for tissue factor, vascular endothelial growth factor, and osteopontin (× 1,000).

No differences were found for gastric and pulmonary tissue sections in our immunohistochemical examinations. All cases showed positive reactivity for TF, five cases showed positive reactivity for VEGF (Case 2, 3, 4, 5, and 6), and three cases showed positive reactivity for OPN (Case 3, 5, and 6) [Figure 6 and Table 4].

**Table 4:** Immunohistochemical examinations of gastric and pulmonary tissue sections in each pulmonary tumor thrombotic microangiopathy case.

	TF		VEGF		OPN	
Case	Gastric lesion	Pulmonary lesion	Gastric lesion	Pulmonary lesion	Gastric lesion	Pulmonary lesion
1	+	+	-	-	-	-
2	+	+	+	+	-	-
3	+	+	+	+	+	+
4	+	+	+	+	-	-
5	+	+	+	+	+	+
6	+	+	+	+	+	+

TF: Tissue factor, VEGF: Vascular endothelial growth factor, OPN: Osteopontin

No differences were found for gastric and pulmonary tissue sections in our immunohistochemical examinations. All cases showed positive reactivity for tissue factor, five cases showed positive reactivity for vascular endothelial growth factor (Case 2, 3, 4, 5, and 6), and three cases showed positive reactivity for osteopontin (Case 3, 5, and 6).

## Discussion

PTTM has been regarded as a rare and severe devastating cancer-related pulmonary complication, the majority of which is mainly induced by poorly differentiated adenocarcinoma arising from gastric mucosa [2]. Although it has been well known that the *ante mortem *diagnosis of PTTM is very difficult [6], Miyano et al. reported a PTTM case which could be determined through an *ante mortem *diagnosis and the patient survived after chemotherapy [9]. This report emphasized the necessity and importance of intensive monitoring and prophylactic intervention, especially in the case of advanced gastric carcinoma, in order to make an early diagnosis and save the life of patients with PTTM.

According to previous research [1,2], the incidence of PTTM in autopsy cases with gastric carcinoma has been reported ranging from 16.7% (6 PTTM cases among 36 gastric carcinoma patients) to 26.8% (11 PTTM cases among 41 gastric carcinoma patients). Similarly, our study revealed an incidence of 16.2% (6 PTTM cases among 37 gastric carcinoma patients), which is largely consistent with these previous reports. In addition, the most common histological type of gastric carcinoma in the present study was poorly differentiated adenocarcinoma (five of the six cases). This fact is also consistent with the previous studies which reported that the most common tumor associated with PTTM is poorly differentiated adenocarcinoma [1,2].

We now wish to discuss PTTM in more detail from the viewpoint of our morphometric analysis of pulmonary arteries. Since some patients with PTTM suffered from pulmonary hypertension, we suggested that pulmonary arterial remodeling induced by carcinoma cell adhesion onto the endothelium affected the status of pulmonary hypertension. Therefore, in the present study, morphometric analysis was carried out on pulmonary arteries. Results showed that the degree of luminal narrowing of the pulmonary arteries varied from case to case. We focused particularly on case 1 and 2 because pulmonary arterial pressure was evaluated only in these two cases. Case 1 involved clinically proven pulmonary hypertension and revealed widespread severe stenosis in the pulmonary artery that showed a significant negative association between arterial diameter and luminal stenosis rate, as well as pulmonary arterial hypertension as reported by Heath and Edwards [1]. In contrast, case 2 involved clinically denied pulmonary hypertension and showed a significant negative association between arterial diameter and luminal stenosis rate. However, its stenosis was milder and involved a smaller artery in comparison to case 1. Accordingly, the occurrence of pulmonary hypertension may require a widespread pulmonary lesion with severe luminal narrowing. That is, pulmonary hypertension in a patient with PTTM may occur in the case of which distribution of pulmonary lesion is similar to that shown by a pulmonary hypertension case. Then, we prefer to make a little discussion on the histopathological difference between PTTM and pulmonary arterial hypertension (PAH). The initial event of PAH is understood as luminal stenosis due to a symmetric intimal or medial thickening involving a terminal arteriole with a diameter less than 100 μm [8]. However, quite interestingly, our statistical analysis revealed no significant difference for average stenosis rate between the under 100-μm group and the 100 to 300-μm group in the all PTTM cases of this study. These results suggest that carcinoma cell adhesion onto the endothelium in PTTM tends to start from larger arteries rather than a terminal pulmonary arteriole.

Incidentally, VEGF and/or TF expression by carcinoma cells has been confirmed in many cases of PTTM [2-7]. VEGF has been known as an endothelial cell-specific angiogenetic mitogen and is upregulated by TF [2]. Furthermore, Takahashi et al. reported a case of PTTM with OPN expression [7] and they suggested that OPN promotes thrombus formation, local activation of coagulatory events, and pulmonary hypertension in the pathogenesis of PTTM. Therefore, we also performed immunohistochemical examinations of both gastric and pulmonary tissue sections from each patient. In the present study, 100% (6/6), 83.3% (5/6) and 50.0% (3/6) of PTTM cases showed positive reactivity for TF, VEGF, and OPN, respectively. It has been reported that 25.1% (52/207) [10], 76.4% (113/148) [11], and 69.5% (205/295) [12] of gastric carcinoma cases show positive reactivity for TF, VEGF, and OPN, respectively. Accordingly, our immunohistochemical examination indicated that gastric carcinoma of PTTM shows a higher TF-positive rate than typical gastric carcinoma. However, it still remains obscuring whether gastric carcinoma inducing PTTM shows a higher immunohistochemical positive rate for VEGF or OPN because typical gastric carcinoma also shows a high immunohistochemical positive rate for these entities. Moreover, no significant difference was found in characteristics of phenotypic expression of VEGF or OPN for gastric adenocarcinoma cells between those with and without PTTM, which represents an unknown and important factor that may influence the development of PTTM.

## Conclusion

In the present study, we examined six cases of PTTM, histopathologically, and revealed that the degree of luminal narrowing of the pulmonary arteries varied from case to case. Our results suggested that pulmonary hypertension in PTTM occurs in selected cases which have a widespread pulmonary lesion with severe luminal narrowing in the smaller arteries. Furthermore, the immunohistochemical examination indicated that gastric carcinoma inducing PTTM shows a higher TF-positive rate than typical gastric carcinoma. However, it still remains obscuring. Further investigations should be required to know whether gastric carcinoma inducing PTTM shows a higher VEGF or OPN-positive rate as determined by immunohistochemistry.

## Abbreviation

PTTM: pulmonary tumor thrombotic microangiopathy; TF: tissue factor; VEGF: vascular endothelial growth factor; OPN: osteopontin; HE: hematoxylin and eosin; EVG: Elastica van Gieson; IELA: area within the internal elastic lamina; LA: lumen area; ANOVA: analysis of variance; SD: standard deviation; PAH: pulmonary arterial hypertension.

## Consent

Written informed consent (in Japanese) was obtained from the patients' family for publication of this study and any accompanying images. A copy of the written consent is available for review by the Editor-in-Chief of this journal.

## Competing interests

Dr. Shibuya reports receiving research grants from Pfizer Inc., Janssen Pharmaceutical K.K., and Dainippon Sumitomo Pharma Co. All authors declare that they have no competing interests.

## Authors' contributions

YO conceptualized this study, integrated the data, and wrote the manuscript as a major contributor; MW carried out the histopathological evaluation and revised the manuscript; KK carried out statistical evaluation and revised the manuscript; TN carried out the histopathological evaluation and gave final approval to the manuscript as a corresponding author; TY carried out a part of the histopathological and statistical evaluation; FA, NH, and DS searched and extracted data from autopsy records in the Toho University Omori Medical Center and photographed a part of the pulmonary arterial images; MS, HN, and TI carried out the HE, EVG, and immunohistochemical stain, and a part of statistical evaluation; KS carried out histopathological and statistical evaluation and revised the manuscript as a last author. All authors contributed to conceptualizing and writing this study. Furthermore, all authors read and approved the final manuscript.
